# Reconstruction using a frozen autograft for a skull and humeral lesion of synchronous multicentric osteosarcoma after undergoing successful neoadjuvant chemotherapy: a case report and review of the literature

**DOI:** 10.1186/s12893-020-01018-w

**Published:** 2021-01-22

**Authors:** Yoshihiro Araki, Katsuhiro Hayashi, Norio Yamamoto, Akihiko Takeuchi, Shinji Miwa, Kentaro Igarashi, Takashi Higuchi, Kensaku Abe, Yuta Taniguchi, Hirotaka Yonezawa, Sei Morinaga, Yohei Asano, Takayuki Nojima, Hiroyuki Tsuchiya

**Affiliations:** 1 Department of Orthopaedic Surgery, Graduate School of Medical Sciences, Kanazawa University, 13-1, Takaramachi, Kanazawa, Ishikawa 920-8641 Japan; 2 Department of Pathology, Kanazawa University, Kanazawa, Japan

**Keywords:** Synchronous multicentric osteosarcoma, Skull lesion, Bone scan, Chemotherapy, Total necrosis, Frozen autograft, Background

## Abstract

**Background:**

Synchronous multicentric osteosarcoma (SMOS) is a rare disease characterized by simultaneous multicentricity of intraosseous osteosarcoma without visceral involvement. SMOS, including a skull lesion, which occurs relatively rarely, and reconstruction using a frozen autograft after the excision of a lesion of SMOS has been infrequently reported previously.

**Case presentation:**

We report an 18-year-old girl with SMOS, with lesions located in the left distal femur, right proximal humerus, and left occipital bone. Her major complaint was pain and swelling around the left knee joint. Asymptomatic lesions of the humerus and skull bone were detected on a systemic bone scan. No visceral organ metastasis was observed. A biopsy of the distal femoral lesion revealed osteosarcoma. Based on the histological findings, multiple bone lesions, and absence of visceral lesion, the clinical diagnosis of SMOS was made. After five courses of neoadjuvant chemotherapy with a regimen of doxorubicin and cisplatin, reconstruction using a tumor prosthesis following wide excision of the left distal femur was performed, and total necrosis was histologically observed in the retracted specimen. Following three cycles of adjuvant chemotherapy, tumor excision and reconstruction with a frozen autograft treated with liquid nitrogen was conducted for both lesions of the humerus and skull, rather than tumor prosthesis or synthetics, in order to retain a normal shoulder function, and to obtain a good cosmetic and functional outcome after treatment of the skull lesion. Further adjuvant chemotherapy could not be administered after the completion of the surgical treatment for all lesions because the adverse events due to chemotherapy were observed. At over 5 years after the diagnosis, she remains clinically disease-free.

**Conclusions:**

An early correct diagnosis, the proper management of chemotherapy, and surgical treatment for all lesions are essential for achieving a good clinical outcome, even in SMOS including a skull lesion. By performing reconstruction using a frozen autograft for a proximal humeral lesion and a skull lesion after confirming the good histological efficacy of neoadjuvant chemotherapy for the primary lesion, the excellent function of the shoulder joint and a good cosmetic outcome at the site of the skull lesion was acquired without complications or recurrence.

Synchronous multicentric osteosarcoma (SMOS) is characterized by simultaneous multicentricity of intraosseous lesion without visceral involvement, such as lung metastasis [1]. SMOS is a rare disease that accounts for approximately 1% of osteosarcomas, and had been reported to have a poor survival because of the difficulty of treating all lesions [2–7]. Domenico et al. reported that the 2-year overall survival of SMOS was approximately 30%, and the 5-year overall survival was < 10% [2]. There have been a few reports regarding the long-term survival of SMOS patients [2, 5, 6, 8]. No standard treatment algorithm has been previously reported because of its rarity, and most cases were treated according to the general guidelines for the management of osteosarcoma.

Osteosarcoma at the skull region is also rare, and acounting for only 6 to 8% of osteosarcomas [3, 9]. The mortality was reported to be about 50% at 5 years [9, 10]. The poor outcome was reported to be due to the difficulty of surgical treatment with a wide excision due to the complicated anatomy of the skull [10–12]. In most cases, synthetics or no reconstruction was selected after the excision of skull lesion. However, complications such as failure of synthetics, or infection, and local relapse of skull osteosarcoma often leads to severe functional disorders due to brain damage, such as extremity paralysis and meningitis, although metastases to the lung and brain are less frequent than extremity osteosarcoma [7, 13, 14].

Chemotherapy is very important for supporting the surgical outcome of tumor patients. In a few previous reports, long survivors of SMOS also had necessarily received chemotherapy, and more than 90% necrosis of the tumor cells in the specimen excised from the patients had been observed histologically [5, 7, 8]. Aggressive surgery with a clear margin for all lesions of SMOS is also essential for a good oncological outcome [5, 7, 8].Thus, planning of an appropriate strategy for the treatment of SMOS, which combines chemotherapy and surgery, is essential for obtaining a good clinical outcome (Fig. 1).

**Fig. 1:**
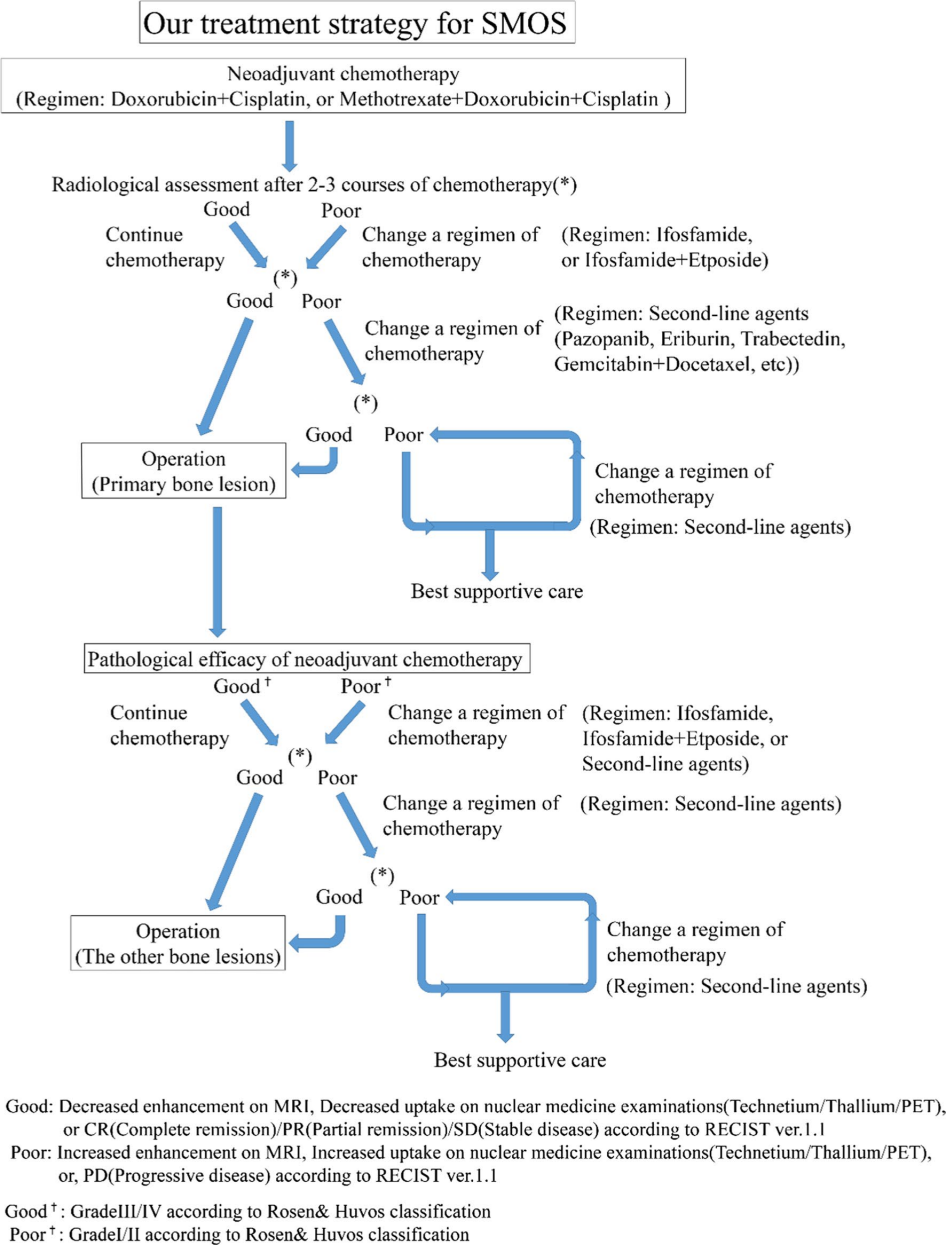
Our treatment strategy for synchronous multicentric osteosarcoma (SMOS)

We herein report an 18-year-old girl with multicentric synchronous osteosarcoma, including a skull lesion, and describe the clinical findings and treatment outcome for chemotherapy, two-stage tumor excision for all lesions. Our study was approved by the ethics committee of Kanazawa University Hospital (Institutional Review Board (IRB) number 2019–61(3094)) in compliance with the guidelines of the 1975 Declaration of Helsinki. Written informed consent was provided by the patient to obtain her case details and any accompanying images published.

## Case presentation

**Fig. 2:**
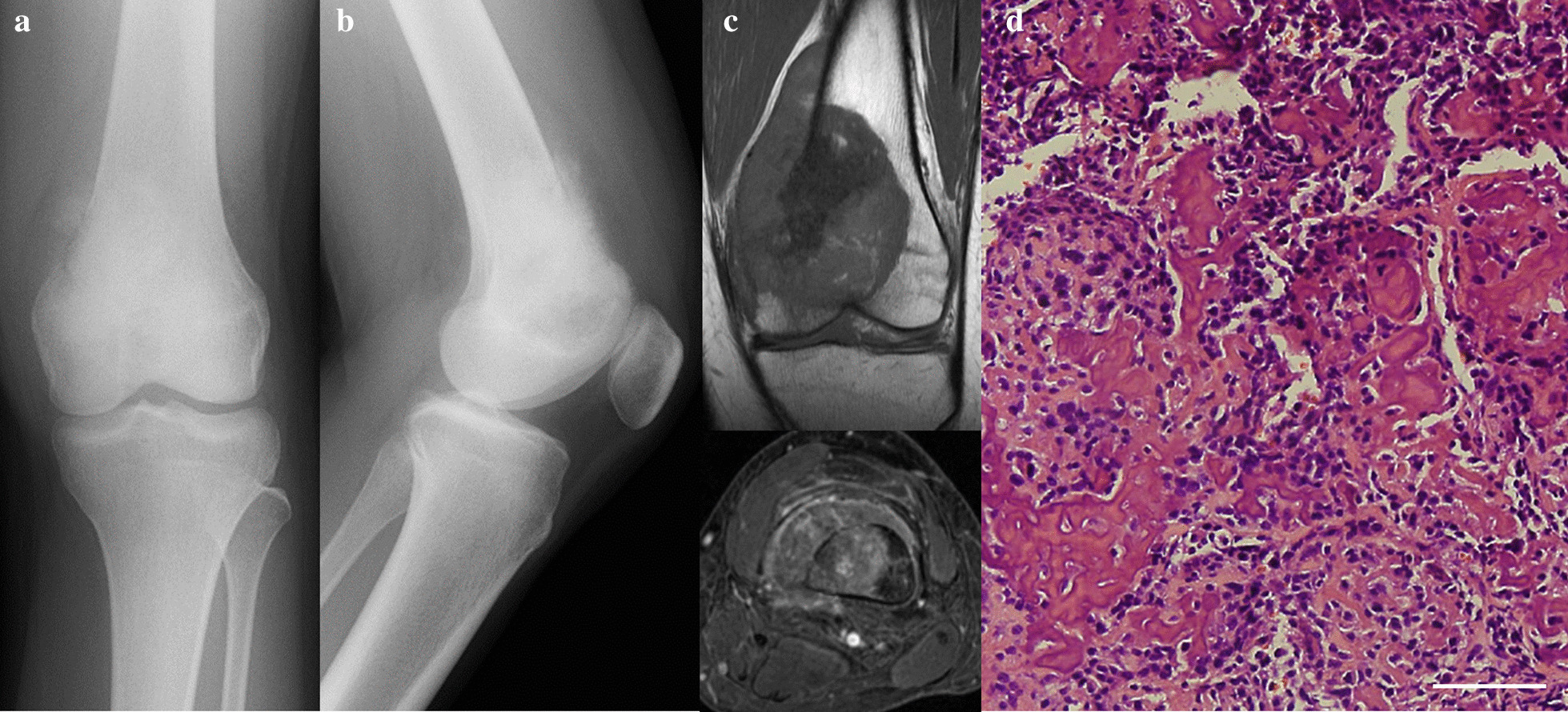
Radiological and pathological findings of a bone lesion at the left distal femur. **a**, **b** Preoperative roentgenogram on the anteroposterior view (**a**), and the lateral view (**b**). **c** Intraosseous lesion combined with extraosseous lesion on MRI was observed, which was hypointense signal on T1 weighted images, and hyperintense signal on T2 weighted images. **d** Enhanced MRI revealed unevenly contrasted lesion on axial images. **e** Proliferation of pleomorphic spindle cells with nuclear atypia and brisk mitosis, producing a large amount of lace-like osteoid, were observed. White scale bar shows 100 µm

**Fig. 3:**
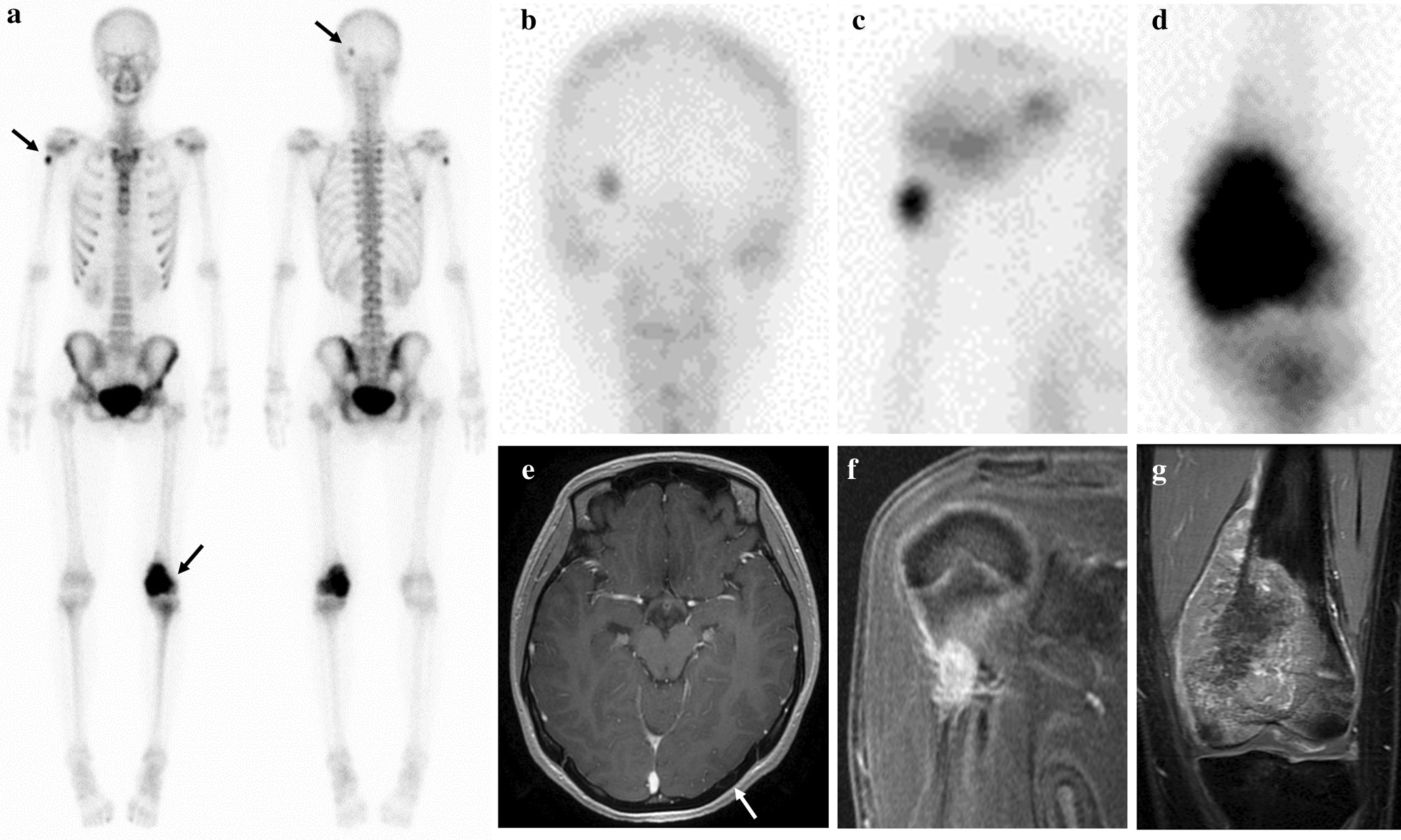
Systemic bone scan and MRI findings before neoadjuvant chemotherapy. **a**–**c** Bone scan revealed uptake for the following three location (**a**); left occipital bone (**b**), right proximal humerus (**c**), and left distal femur (**d**). **e** The left occipital lesion on axial enhanced MRI, and a white arrow shows the intracortical lesion. **f** The right proximal humeral lesion on coronal enhanced MRI. **g** The left distal femoral lesion on coronal enhanced MRI

An 18-year-old woman was referred to our department with a major complaint of a pain and swelling around the left distal femur. The symptom had been increasing for the past 6 months. She consulted a local doctor because her symptoms had not improved over time. Neither trauma nor injury was reported. Her previous history was also unremarkable, and no family history was found. A physical examination revealed swelling around her left distal femur and tenderness upon palpation. Neither ballottement of the left knee joint nor an evident soft tissue mass were observed. Laboratory tests revealed a threefold higher alkali-phosphatase level than the upper limit, but no other abnormal data, including the lactate dehydrogenase level and inflammation reaction, was found. A bone sclerotic lesion with periosteal reaction on the distal femur was found on X-ray (Fig. 2a, b). Computed tomogram (CT) of the left distal femur revealed a sclerotic and lytic lesion causing partial cortical destruction with soft tissue extension to the medial part of the surrounding muscles. No distant metastasis, including to the lung and regional lymph nodes, were observed on chest or abdominal CT. Magnetic resonance imaging (MRI) showed an intraosseous lesion combined with an unclear circumscribed extraosseous mass, which was hypointense on T1-weighted (Fig. 2c) and hyperintense on T2-weighted images. Unevenly contrasted lesion was observed on enhanced MRI (Fig. 2d). An open biopsy for the left distal femoral lesion was performed. Proliferation of pleomorphic spindle cells with nuclear atypia and brisk mitosis, producing a large amount of lace-like osteoid, were observed (Fig. 2e). A systemic Technetium-99 m scintigraphy (bone scan) (Fig. 3a) revealed other bone lesions of the left occipital bone (Fig. 3b) and right proximal humerus (Fig. 3c), in addition to the left distal femoral lesion (Fig. 3d), although no evident physical findings on either the right proximal humerus or left occipital bone were observed. However, a relatively strong uptake was observed at all three lesions. Thallium scintigram also revealed the strong accumulation of tracers only on the three bone lesions. A close examination by MRI revealed the enhancement of right proximal humeral bone lesion and left occipital bone lesion (Fig. 3e, f), the same as the left distal femoral lesion (Fig. 3g). Based on the histological findings, multiple bone lesions, and absence of visceral lesion, the clinical diagnosis of synchronous multicentric osteosarcoma (SMOS) was made. There is much debate in the previous studies as to whether it represents multiple primary tumors or metastatic disease. However, the case for multiple primary tumors was favored, because there was no obvious route for spread if the lungs were tumor-free, which was thought to rule out hematogenous metastasis. The prognosis of SMOS is generally considered to be poor according to the findings of previous studies [2–7].

**Fig. 4:**
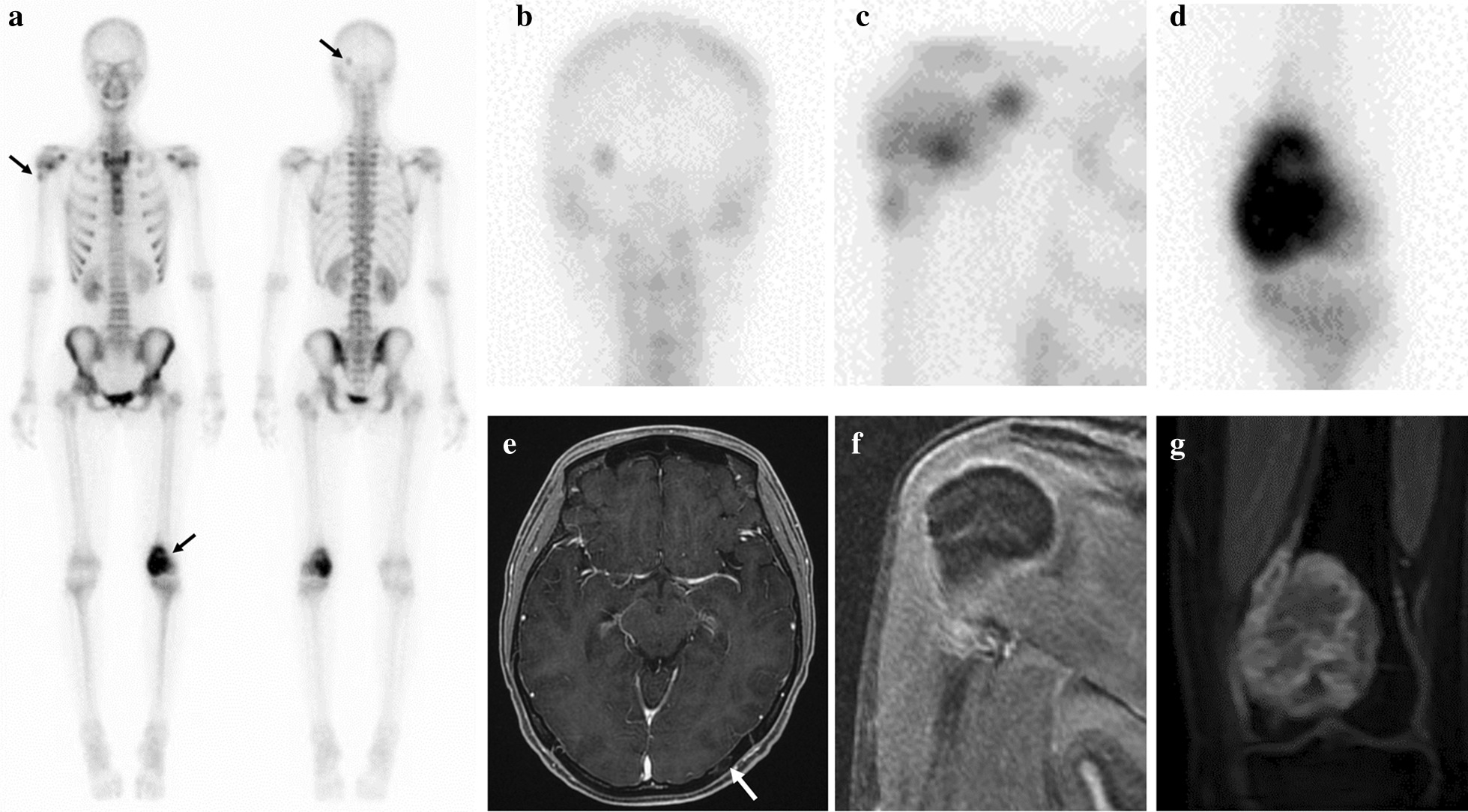
Systemic bone scan and MRI findings after five courses of neoadjuvant chemotherapy. **a** The uptake of all lesions on the bone scan was diminished. **b** Occipital bone lesion. **c** Right proximal humeral lesion. **d** Left distal femoral lesion. **e** The left occipital lesion had slightly shrunk in size on axial enhanced MRI (a white arrow shows the intracortical lesion.). **f** The enhancement was drastically decreased on coronal MRI of the right proximal humeral lesion. **g** The size and enhancement was decreased on coronal MRI of the distal femur

She underwent five courses of neoadjuvant chemotherapy with a regimen of doxorubicin and cisplatin, which was the same as the standard regimen for the treatment of osteosarcoma. The dose of doxorubicin was 60 mg/m^2^, and that of cisplatin was 120 mg/m^2^. The chemotherapy was performed every three weeks. After the completion of neoadjuvant chemotherapy, the uptake of all lesions on the bone scan diminished (Fig. 4a–d), and the enhancement of all lesions on MRI decreased (Fig. 4e–g). The accumulation of tracers on thallium scintigram also decreased. The chemotherapy was observed to be highly effective based on the radiological findings. The alkali-phosphatase level dramatically decreased and become normal in laboratory tests.

**Fig. 5:**
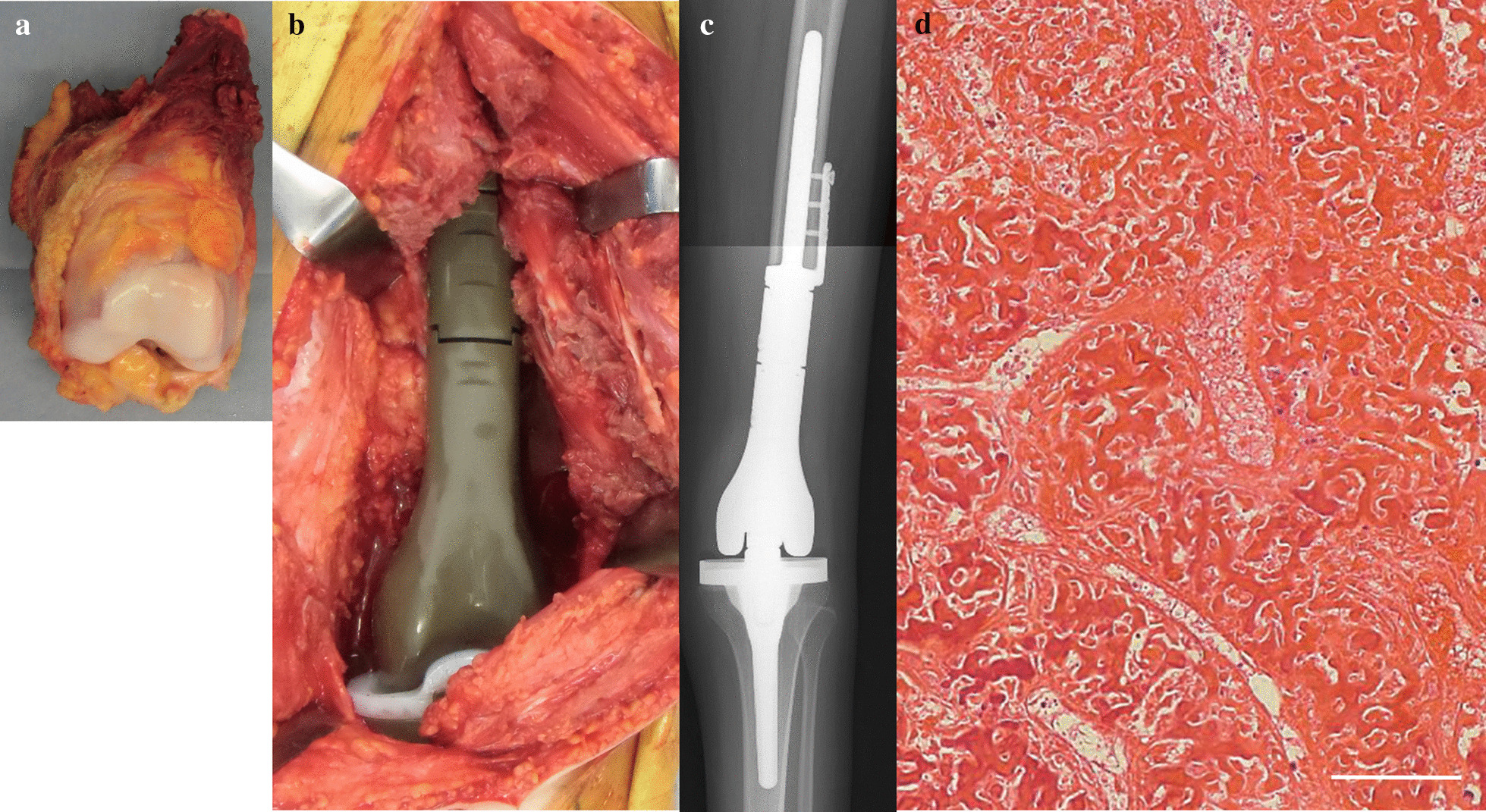
Intraoperative photos, the postoperative roentgenogram, and the histological findings of the retracted specimen. **a** Wide excision of the left distal femoral lesion was performed. **b** Reconstruction using iodine-coated tumor prosthesis was performed. **c** Postoperative roentgenogram. d. Histological findings revealed the total necrosis of tumor cells of the retracted specimen tissue of the distal femur. White scale bar shows 100 µm

Surgical treatment of wide excision for the primary lesion of the left distal femur and reconstruction with megaprosthesis was planned at 4 months after the diagnosis. The distal femoral lesion was widely excised with extraosseous lesion and biopsy tract, preserving popliteal artery, veins and sciatic nerve (Fig. 5a). The bone was cut at 3 cm proximal from the edge of the bone lesion. A portion of the vastus medialis and intermedius were excised along with the tumor. The iodine-coated tumor prosthesis [15–17] was used for reconstruction (Fig. 5b, c). The pathological findings for the excised specimen revealed total necrosis of the lesion (Fig. 5d), and free surgical margins for both the bone and soft tissue lesions of the left distal femur.

Since physical recovery from the primary surgery and wound healing were observed, adjuvant chemotherapy was resumed at seventeen days postoperatively for the residual lesions of the humerus and occipital bone. The regimen for an adjuvant chemotherapy was the same as a neoadjuvant chemotherapy. A mildly decreased cardiac function was observed on echocardiogram after the completion of the first course of adjuvant chemotherapy, and chemotherapy with another regimen of high-dose methotrexate (10 g/m^2^) and vincristine (2 mg/body) was performed for the second course of adjuvant chemotherapy. The improvement of cardiac function was confirmed, and the doxorubicin (60 mg/m^2^) and cisplatin (96 mg/m^2^, 80% dose of standard regimen) were given again for the third course of adjuvant chemotherapy. A total of eight courses of chemotherapy were completed, including three courses of adjuvant chemotherapy. The total dose of doxorubicin and cisplatin was 420 mg/m^2^ and 816 mg/m^2^, respectively. The humeral lesion had almost completely disappeared radiologically, and the occipital lesion was only slightly visible on images. Both the alkali-phosphatase level and the lactate dehydrogenase level remained normal in laboratory tests.

**Fig. 6:**
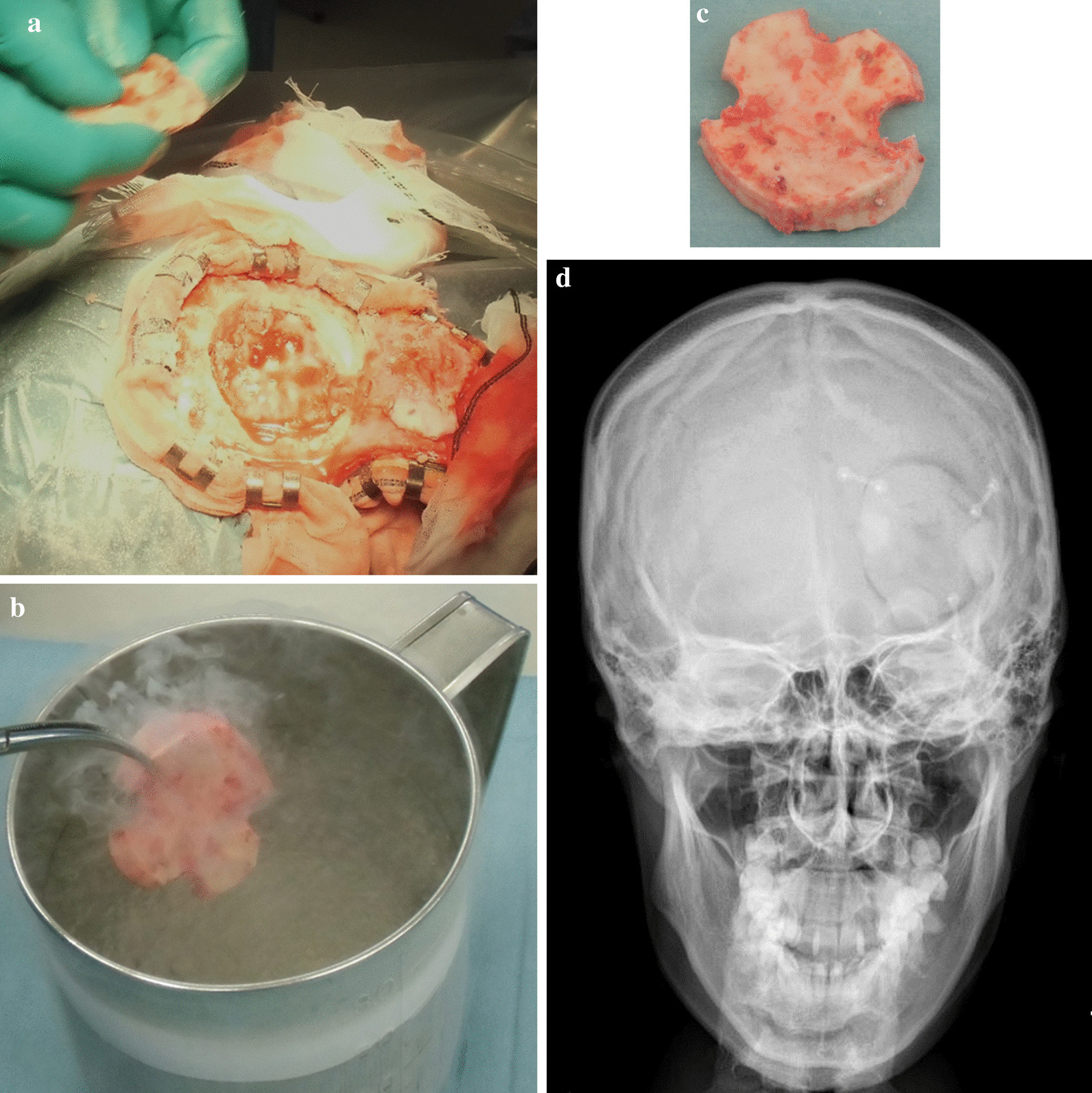
Surgical treatment for a skull lesion and the postoperative roentgenogram. **a** Skull lesion was excised with wide margin. **b**, **c** The excised bone was treated with liquid nitrogen for twenty minutes (**b**), and dissolved at room temperature for 15 min, and then was washed with 0.3% iodine saline and distilled water (**c**). **d** The frozen bone was returned to the original position and plate fixation was performed. Postoperative roentgenogram was shown

**Fig. 7:**
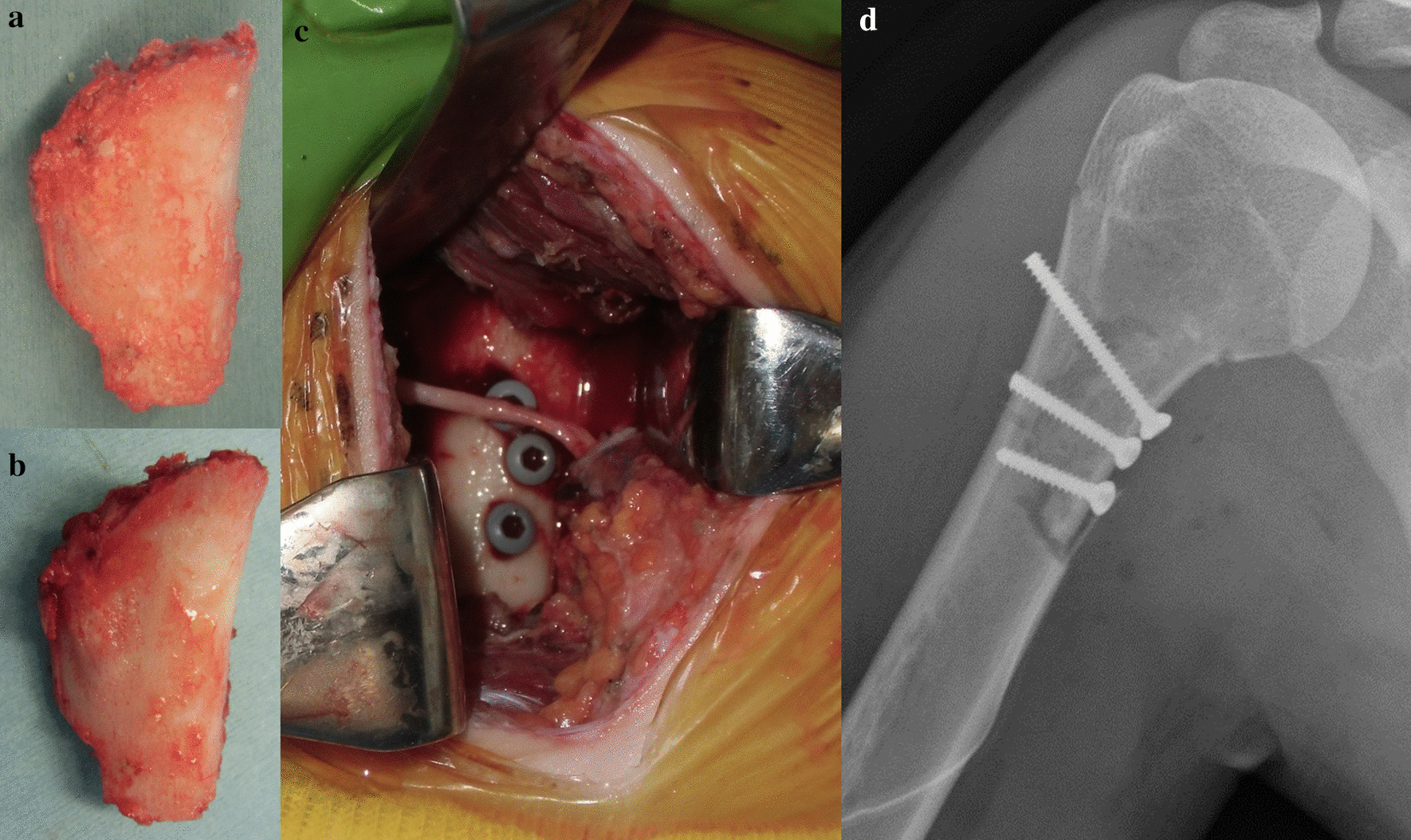
Surgical treatment for a proximal humeral lesion and the postoperative roentgenogram. **a**, **b** The retracted hemicortical bone of the proximal humeral lesion was treated with liquid nitrogen (**a**) and was dissolved at room temperature, and washed by 0.3% iodine saline and distilled water (**b**). **c** The frozen autograft was returned to the original position with screw fixation, preserving axillary nerve. **d** Postoperative roentgenogram was shown

Tumor excision of both the humeral lesion and the occipital lesion, and reconstruction using a frozen autograft, rather than a tumor prosthesis or synthetics, was planned at 6 months after the diagnosis, because a histological analysis demonstrated that chemotherapy showed good efficacy in the treatment of the primary lesion. For the humeral lesion, the tumor location was identified using a fluoroscopy, based on preoperative MRI findings. For the skull lesion, the tumor location was identified with the assistance of a navigation system. At first, tumor excision for the occipital lesion was performed with a margin of at least 2 cm (Fig. 6a), and the excised bone was treated with liquid nitrogen for 20 min (Fig. 6b). The frozen autograft was dissolved at room temperature for 15 min, and washed by 0.3% iodine saline and distilled water (Fig. 6c). The autograft was returned to the original position with plate fixation (Fig. 6d). Then, hemicortical excision for the right proximal humeral lesion was performed with a margin of at least 2 cm, preserving axillary nerve, and then the excised bone was treated with liquid nitrogen for 20 min (Fig. 7a). The frozen autograft was dissolved at room temperature for 15 min, and washed by 0.3% iodine saline and distilled water (Fig. 7b). The autograft was returned to the original position with screw fixation (Fig. 7c, d).

Further chemotherapy was not administered after the second surgery because the adverse events due to chemotherapy, such as a myelosuppression and a mildly-decreased cardiac function, were observed. She was discharged from the hospital after the recovery from the surgery was confirmed. She was prudently observed under active surveillance, and underwent close follow-up examinations in an outpatient clinic every 3 or 4 months after discharge.

**Fig. 8:**
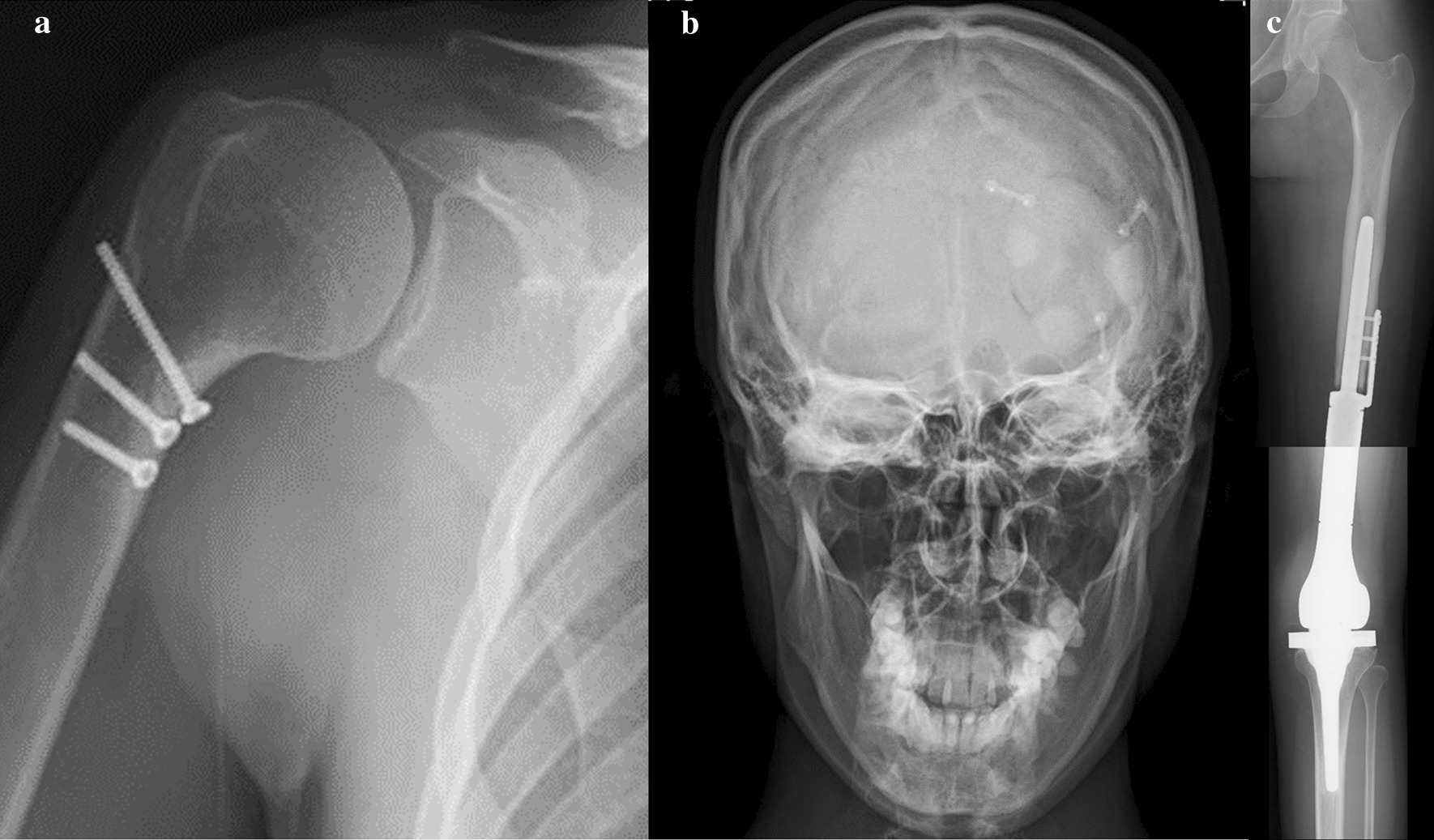
The postoperative roentgenogram at the latest follow-up. **a** Bone union at the osteotomy site for proximal humeral lesion, was completely achieved. **b** Bone union at the osteotomy site for skull lesion, was achieved. **c** Postoperative complications were not observed after tumor prosthesis reconstruction of the left distal femur

At 68 months’ follow-up after the detection of SMOS, a disease-free condition with neither local recurrence nor metastasis was obtained, and bone union for the humerus and skull lesion had also been achieved on images (Fig. 8a–c). The patient’s cardiac function was normal on an echocardiogram, and no other adverse events was observed at the last follow-up. Both the alkali-phosphatase level and the lactate dehydrogenase level remained normal in laboratory tests. An excellent function for both the upper extremity (30/30 points) and lower extremity (30/30 points) according to the International Society of Limb Salvage (ISOLS) score had been acquired. No neurological deficit was observed.

## Discussion and conclusions

Multicentric osteosarcoma accounts for only approximately 1% of all osteosarcomas, and is divided into two types: synchronous and metachronous type [2, 3]. Domenico et al. reported that the median survival in patients with metachronous multicentric osteosarcoma (n = 34) was 43 months, while the median survival in those with SMOS (n = 22) was 14 months [2]. A particular poor prognosis was reported for SMOS patients, and there have been few previous report concerning long-term survivors of SMOS [2, 5, 6, 8]. To our knowledge, the survival of SMOS, including cases with skull lesions, tends to be extremely short (4–18 months) [7, 13, 14]. There were three main reasons for the successful outcome of the present case. First, the early correct identification of tumors at all locations could be made using a bone scan. Second, multiple chemotherapeutic agents, including doxorubicin and cisplatin, would contribute to the good response of the tumors, as histological examinations revealed complete necrosis in the excised specimen of the distal femur in our case. Third, surgical treatment for the other lesions could be performed with the reference to the results of neoadjuvant chemotherapy, which showed histological efficacy.

Our treatment strategy for SMOS is basically the same as that for osteosarcoma (Fig. 1). To identify precisely all of the locations of SMOS lesions before treatment is important for observing the treatment course. The good efficacy of chemotherapy is essential for obtaining good outcome. After the completion of neoadjuvant chemotherapy, a radiological assessment is performed to determine the efficacy of chemotherapy. If the course is assumed to have poor efficacy based on radiological assessment, the chemotherapy regimen should be changed. When good efficacy is radiologically confirmed after five or six courses of neoadjuvant chemotherapy, subsequent surgical excision of the primary lesion, with a wide margin, is planned and performed. Then, based on the pathological assessment of the efficacy of neoadjuvant chemotherapy in the excised specimen, an adjuvant chemotherapy regimen is selected. If the neoadjuvant chemotherapy shows good histological efficacy, three or four courses of adjuvant chemotherapy with the same regimen are performed, and an appropriate surgical treatment for the residual lesions is planned, including biological reconstruction, which can retain a normal function and achieve a good cosmetic outcome. If the histological efficacy of neoadjuvant chemotherapy is poor, the regimen should be changed for adjuvant chemotherapy, and surgical treatment with as wide an excision as possible and reconstruction using tumor prosthesis or synthetics, should be planned after several courses of chemotherapy.

When the surgical margins for all lesions are histologically clear, the primary treatment is considered to be over, or additional adjuvant chemotherapy may be considered if the patients can afford to undergo these treatments, with careful observation for adverse events. After completion of the primary treatment, the postoperative state will be prudently observed at follow-up in an outpatient clinic, with examinations every three or four months until at least five years at least after surgery.

Osteosarcoma commonly presents with lung metastases, and a standard examination includes chest CT. Additional examinations are not regularly performed except for when a patient presents symptoms, such as pain or swelling. Patients with skull osteosarcoma often present with headache, motor impairment, or cranial nerve palsies, depending on the tumor site [9–12]. However, in our case, no symptoms at the left occipital region or the right proximal humerus were observed, and it was not until a bone scan was performed that multicentric bone lesions were noted. A bone scan can assess the accumulation of tracers, thereby reflecting remodeling of the affected bone, and a systemic bone scan is useful as a regular examination for the early detection of other bone lesions [18], even if no symptoms are observed. Metachronous type multicentric osteosarcoma, or bone metastases of SMOS during the treatment course, might be due to tiny lesions with symptoms mild enough to be overlooked at the first visit. Therefore, the early correct identification of tumors at all locations is important when considering the strategy for SMOS.

Chemotherapy is very important for supporting the surgical outcome of tumor patients. In a few previous reports, long survivors of SMOS had necessarily received chemotherapy, and more than 90% necrosis of the tumor cells in the specimen excised from the patients had been observed histologically due to the efficacy of chemotherapy, which was assessed according to the Rosen and Huvos classification [5, 7, 8]. One patient received chemotherapy with a regimen of doxorubicin, cisplatin, ifosfamide, and methotrexate, and another received chemotherapy with a regimen of doxorubicin, cisplatin, and methotrexate [5, 7], while yet another patient received chemotherapy with a regimen of methotrexate, ifosfamide, bleomycin, doxorubicin, and cisplatin [8]. The present patient received chemotherapy with a regimen of doxorubicin, cisplatin, methotrexate, and etoposide. The two drugs, namely doxorubicin and cisplatin, have been commonly used for long survivors in previous SMOS patients including our case. Multiple chemotherapeutic agents including the above-mentioned two drugs might contribute to the good outcome observed in SMOS patients, although no definitive conclusions could be made due to the small sample population.

The histological confirmation of the efficacy of neoadjuvant chemotherapy in the whole section of the distal femoral lesion, especially the bone lesion, was necessary for determining whether the same regimen chemotherapy for the other two lesions should be continued or not. When a frozen autograft is used for reconstruction after wide excision, the efficacy of chemotherapy on excised soft tissue lesion can be evaluated histologically; however, the efficacy is not evaluated in bone lesion. Frozen autograft is available for almost all bone tumors and has been reported to be associated with many advantages, including preservation of the bone stock, retention of the joint function in cases in which the epiphysis is preserved, and the fact that the graft can be a perfect fit for the excised bone defect. However, the indications need to be well considered in each case. In cases involving multiple bone lesions, such as the present case, the selection of an appropriate treatment strategy is essential, and we referred to the results of a histological assessment of the effects of neoadjuvant chemotherapy on the primary bone lesion when considering treatment for residual lesions. In addition, the epiphysis could not be preserved in the present case because the lesion extended to the epiphysis. Thus, a tumor prosthesis was selected for reconstruction after wide excision of the distal femoral primary lesion.

Hemicortical excision with a clear margin and reconstruction using a frozen autograft treated with liquid nitrogen is reportedly a reliable technique for retaining a good joint function through minimally resection of the tumor and preservation of as much of the normal tissue around the resected tumor as possible [19–25]. This procedure was applied in our case because the neoadjuvant chemotherapy showed good histological efficacy for the primary lesion. In our case, an excellent right shoulder joint function was restored, and a bone union was achieved, and no recurrence or postoperative complications were observed (Fig. 8a).

Complete excision with a wide margin was associated with an improved survival, and local recurrence after surgery of the skull tumor is the major cause of treatment failure and decreases the survival rate. However, the anatomy of the head is complicated, and complete resection of the skull lesion is often difficult to achieve [10–12]. Synthetic or plate reconstruction after tumor excision is generally performed to protect the brain and fill bone defects, but there are sometimes cosmetic issues, and the complications, such as infection, or motor impairment due to brain damage, cannot be denied. Tumor excision of skull lesions and reconstruction using a frozen autograft treated with liquid nitrogen have been rarely reported [26–28]. However, this cryotherapy treatment and orthotopic transplantation method not only eliminates the tumor cells, but it also provides for bone filling which perfectly matches the same size as the bone defect, while also reducing the incidence rates of complications [19, 20, 26–28]. The good histological efficacy of neoadjuvant chemotherapy for the primary lesion was observed in our case; thus, reconstruction using a frozen autograft treated with liquid nitrogen after tumor excision with a clear margin, was applied. After surgery, a bone union was achieved without postoperative complications or local recurrence (Fig. 8b).

In conclusion, SMOS including a skull lesion is rare; nevertheless, the early, correct diagnosis, and proper strategy of chemotherapy and surgery for all lesions, are essential for ensuring a good clinical outcome. Reconstruction using a frozen autograft treated with liquid nitrogen was found to be feasible for bone lesions, including a skull lesion, even in a case of multicentric osteosarcoma, when the histological efficacy of neoadjuvant chemotherapy for the primary lesion was good. Our patient achieved an excellent right shoulder function, and a good cosmetic and functional outcome without any complications by reconstruction by following reconstruction using a frozen autograft. In addition, clinically disease-free survival of over five years after the diagnosis can be obtained, although further follow-up with regular close examinations will be required, as the survival associated with this disease is commonly poor.

## Data Availability

All data generated or analyzed during the present study are included in this published article.

## Abbreviations

SMOS: Synchronous multicentric osteosarcoma
CT: Computed tomogram
MRI: Magnetic resonance imaging
ISOLS: International society of limb salvage
